# Report of an Interesting Case

**Published:** 1906-10

**Authors:** Dunbar Roy

**Affiliations:** Atlanta, Ga.


ATLANTA

### Journal-Record of Medicine

Successor to Atlanta Medical and Surgical Journal, Established 1855, and Southern Medical Record, Established 1870.

OWNED BY THE ATLANTA MEDICAL JOURNAL CO.

**Published Monthly.**

*Official Organ Fulton County Medical Society, State Examining Board, Presbyterian Hospital, Etc.*

VOL. VIII. OCTOBER, 1906. No. 7.

BERNARD WOLFF, M.D., EDITOR, 319-320 Prudential Bldg.

M. B. HUTCHINS, M.D., BUSINESS MANAGER, 1007-1008 Century Bldg.

E. G. BALLENGER, M.D., ASSOCIATE EDITOR AND ASSISTANT MANAGER.

### ORIGINAL COMMUNICATIONS.

### REPORT OF AN INTERESTING CASE. *

BY DUNBAR ROY, M.D.,

ATLANTA, GA.

Mr. E. E., aged fifty-one, cotton merchant. Patient had always been strong and healthy, never even having had a severe spell of illness during his life. Is temperate in both drinking and smoking; in fact, seldom takes a drink. Has lived in Atlanta most of his life. His father and mother are both living, and both in excellent health. In August, 1902, he moved to Shreveport, La., to represent a Northern firm as their cotton buyer. In that city he seemed to enjoy good health, remaining at his usual weight of 150 pounds. Patient was not over four feet nine inches in height. He spent each summer in Atlanta, returning to Shreveport in the fall. During the winter of 1903-04 he did not feel in

* Read before Fulton County Medical Society.

his accustomed good health, and along in the spring of 1904 he began to have neuralgia and drawing of the muscles in the back of the neck. In May, 1904, he returned to Atlanta again to live, having made new connections in the cotton business. On arriving in Atlanta he consulted his physician on account of this severe neuralgic pain in the back of his neck. His appetite was not good and he was already beginning to lose flesh. Being a neighbor and a warm personal friend of mine, I saw him quite frequently, but in an entirely unprofessional manner. In this way I noted his gradual decline from week to week. His physician attributed his trouble to muscular neuralgia, as a result of malaria in his system. He was now treated steadily but without improvement, while the pain gradually extended down into his shoulders and arms. These pains grew to be excruciatingly painful, and would come upon him in paroxysms. His digestion and appetite were poor, and his nightly sleep very much disturbed. Not improving (which is characteristic of a great many patients, and we can hardly blame them) he called in another physician who took charge of his case. He also attributed the cause to malaria, and immediately began to dose him with various medicines, changing the same from time to time. He was put upon a strict diet, and his liver was kept very active. Soon a derangement of the function of this organ was diagnosed as playing a prominent role in producing the various physical ailments. The patient was blessed with an excellent nurse in the shape of his wife, who rubbed and massaged him with various liniments and looked after his every physical and mental comfort.

The pains, however, did not subside, but were extending still further down in patient's back, and even into his legs. At no time were there any signs of paralysis, and the patient was able once a day to go to his office and remain for a few hours.

In giving me the history the patient told me that his physician made a thorough examination of his secretions, urine, etc., and found nothing abnormal. The pains in the shoulders (especially the left) and in the back were sometimes accompanied by such severe spasms of the muscles as almost to draw the head to the shoulder. In September the patient, not improving, but seemingly growing worse, becoming weaker and weaker, losing flesh gradually, and the pains being so severe as to render sleep impossible except for a few hours at night by means of codeine, he consulted a renowned osteopath, who told him it was due to the nerves from the spinal column. He gave him three treatments a week and put him upon a still more restricted diet. This treatment he continued for a month, having discarded all medicines except certain mineral waters suggested by friends. At the end of this time he was no better in any respect, the pains not having been relieved, they being now in the legs as well as in the arms, and accompanied by the most violent spasms. At this point the patient began to be hoarse, but no cough or expectoration.

During a social visit to him one evening he remarked that he had taken a severe cold, and if he didn't get better he was coming to me professionally. In the meantime, up to Christmas, 1904, his wife continued to give him nightly massage, and he would take some of his old medicine to make him sleep. It was frequently remarked among his friends that Mr. E. looked like a dead man, and they believed that he would live but a few weeks. On my return to the city after the Christmas holidays, his wife telephoned to me that Mr. E. wished to consult me in reference to the severe cold in his head and throat. I asked if he was able to come to my office, since I could then give him a more thorough examination and treatment. She said she thought he was able to come down on the car, which he did, being barely able to get to his destination. Being a personal friend and neighbor, I was really sorry that he had come to me for professional advice and treatment, for with his previous history in regard to his medical treatment (of which I was cognizant) I felt that I could do but little for him now that he seemed in the last stages of some obscure nervous disease.

When he came into my consultation room he was so weak, feeble and emaciated that I felt as if he would never be able to get back to his home. His weight then was 108 pounds, having been reduced from 150. He had no signs of paralysis; his reflexes were normal, and his only complaint being the intense pains and spasms in the legs and arms, accompanied with the gradual increased weakness.

Two weeks previous, according to statement, he contracted, in addition to the hoarseness, a cold in the head, and this had gradually kept up until at present it was quite disagreeable, and he had hardly sufficient strength to blow the tough secretion from his nasal cavities. His throat also (i. e., the larynx) worried him considerably by being so tight as to interfere with his breathing, especially at night.

*Examination of Nasal Center.*—At the muco-cutaneous surface in both nostrils, the parts were thickened and inflamed from irritation produced by the nasal discharge. The right cavity seemed pretty well filled with muco-purulent secretion. After shrinking the tissues with cocaine and adrenalin, and by assistance from both patient and myself, the cavity was fairly well cleaned. Far back on both septum and middle turbinal there was a dirty yellowish membrane covering the parts, which extended also into the nasopharynx. A slight manifestation of the same condition was found on the left side, but not quite so marked. The parts bled, some of the membrane was detached. By means of cotton on the end of an applicator I detected roughened and exposed bone high upon the septum at its posterior free border, and also the same condition of the posterior end of the middle turbinal on the same side (i. e., the right). None was detected on the left. The pharynx showed nothing abnormal, but there was a large amount of tough, yellowish muco-purulent secretion coming down from the nasopharynx and clinging to the post-pharyngeal walls. It was impossible to inspect the nasopharynx on account of the relaxed condition of the palate and the gagging produced in my efforts to use a palate retractor.

On inspecting the larynx, I found a beefy-red, swollen condition of the whole laryngeal mucous membrane, the chords being of the same general color. The membrane there and further down in the trachea seemed to be producing a tough, membranous secretion which it was impossible for the patient to raise.

It gave the sound of a "croupy cough." He has no pain either in his throat or nose, but complained some of a pain just at the base of the nose over both frontal sinuses. Transillumination of both frontal sinuses showed no shadow at any point. The objective picture here presented was to me quite significant of a specific condition, but close inquiry elicited not one ray of such a history.

I am sure the patient would gladly have told me if such had been the case, for I told him then that if such was the case he could be improved immediately. I know him intimately, and there were few men who lived a life of more rectitude. Even before his marriage he was never given to the slightest dissipation. The only other condition which such symptoms were likely to represent was tuberculosis, and this, to me, almost seemed the correct diagnosis. I had the secretion from the nose and throat examined, but this was negative of tubercle bacilli. The loss of weight, hoarseness, slight cough, were symptoms quite significant, especially after the negative specific history. However, I knew that histories were ofttimes uncertain, and as an experiment, in conjunction with the local treatment of cleansing and nitrate of silver applications, I put the patient on the following treatment: On account of the poor appetite and atony of the gastric function, he was given ten drops of equal parts of tinct. nux vomica and co. tr. gentian in a little water, and corn whiskey before meals, three times daily. After meals he was started on five drops of a saturated solution iodide potash in one-half glass water, and this was increased one drop every other day. On the morning and afternoon he was given an expectorant mixture containing mixture of carbonate of ammonia, fluid ext. of squills, etc., this being used to soften and aid the expectoration of the tracheal and laryngeal secretion. He was not limited in his diet, but told to eat as much nutritious food as possible. He was also to use a douche of warm saline solution in the nasal cavities three or four times daily.

The next day being very cold, he did not report, but his wife telephoned me that a swelling had developed just at the root of his nose, accompanied by some pain on pressure. In the evening I called to see him and found the patient sitting up in a big chair with a swelling, puffy and globular looking, about the size of a walnut, just at the root of the nose. It had developed slowly during the day. I advised hot fomentations every hour—a good saline internally, and the other treatment to be continued as prescribed. The next day I saw him again, and the edema or swelling had extended to the lids of both eyes until they could not be opened, and even down on the cheek and nose. The original point of the swelling was beginning to subside. The day following I had Dr. Block to see the patient with me in consultation. Dr. Block made a very thorough physical examination of his heart and lungs, muscles, nervous reflexes, and also took a specimen of urine for examination. This latter was found to contain a trace of albumin, but no casts.

Dr. Block was unable to suggest anything further except to give the patient a mixture containing the bromides, to be taken at night for his extreme nervousness. Edema remained limited to the face, and this gradually disappeared entirely in one week's time.

From this time on the patient began to improve, and the internal remedies were never changed, the iodide being increased to fifty drops three times daily. I saw him at my office every other day, cleansed thoroughly the nasal cavity, touched the necrosed condition in the nose and nasopharynx with a strong solution of nitrate of silver, followed finally with a covering of aristol powder. Inhalations of terebene in benzoinated albolin were given for the larynx and trachea. One morning about three weeks after the beginning of the treatment, the patient blew from his nose a mass of secretion and a small piece of bone which seemed to be a part of the vomer. I show you the specimen.

The patient was treated at gradually varying intervals of frequency until August first of this year, i. e., seven months, when he was dismissed apparently cured except that he was still hoarse from the hypertrophied condition of the laryngeal mucous membrane, which, however, is gradually disappearing. I tried astringents and sprays, but this seemed to do but very little good.

The medicine was never changed during the whole course of treatment, the remedies being gradually left off with the improvement of the patient's condition.

December 28, 1905, one year after I first saw him professionally, he weighed 150 pounds, and with the exception of slight hoarseness he is as well and as strong as at any time during his life. There is no discharge in either the nasal cavities or the nasopharynx.

This case presents many interesting as well as obscure features. In the beginning of the patient's trouble, there were some symptoms of a malarial neuritis, and in fact all the physicians consulted during the first months of his illness attributed the cause of his indisposition to malaria. The very first symptom noted by the patient was a general feeling of malaria and loss of appetite. This occurred during the last months of his residence in Shreveport. In May, 1904, he returned to Atlanta, weakened in health and suffering with drawing pains in the muscles at the back of his neck. There was never any rise in the bodily temperature throughout the course of the disease, nor was there at any time any signs of paresis in any portion of the body. It is just possible that a slow grade multiple neuritis might produce some of the symptoms in the case, but my own observation and that of other observers who have written upon this subject would not lead us to such a diagnostic conclusion. For, indeed, at no time during the illness was there signs of paresis, but most prominent were the signs of spasms and intense neuralgia of the sensory nerves.

The diagnosis of malarial intoxication was certainly a most natural one, and it is still a question in my mind whether or not the beginning trouble could not be attributed to this cause.

The brilliant result obtained by the internal administration of the iodide of potash almost compels one to acknowledge the specific basis of the whole constitutional derangement, and yet with the exception of this one fact, i. e., the good results obtained by the internal administration of the iodides, there was absolutely no history or symptoms to warrant us in drawing such a conclusion. I examined him thoroughly at the time, and even since then; there has not been the slightest signs of any glandular enlargement of any portion of the body. No falling out of the hair, no symptoms of sore throat, no obscure pains at night, absolutely nothing to warrant us in suspecting syphilis as the cause. So foreign to my mind was syphilis as the cause of the naso-pharyngeal ulceration at the time of these very active symptoms, that I concluded that the ulceration was without doubt tubercular, coupled with the great loss in weight and the seeming involvement of the bronchial tubes. However, had Dr. Smith to make a most thorough examination of these secretions, and no signs whatever could be found of tubercular bacilli. His mother had been treated for years for a superficial skin cancer on the forehead, and this is still under the constant observation of Dr. Hutchins, which also led me to the tentative belief that the condition in the naso-pharynx might have been of a cancerous nature. However, the complete restoration of these parts compels me to recognize the fallacy of this suspicious diagnosis. Another symptom which was misleading to me was the existence of an odor from the nose in no case characteristic of that experienced in the cases of late tertiary syphilis where the odor is exceedingly offensive, and especially where there are areas of necrosed bone. While many things point to the correctness of our diagnosis, we must remember that the administration of the iodide of potash often proves of benefit and even curative in cases other than syphilis, for as an alternative in various conditions where we can give no better name than "blood dyscrasia" this remedy is often of marked value. As an adjunct to the relief of various inflammatory exudates in the body, I know of no one remedy that is of greater value.

Consequently, because the case responded to the iodide of potash, and seemingly was brought to a successful issue through such administration, is no self-evident proof that we were correct in our diagnosis.

The question could very naturally arise, Was malarial intoxication the primal cause of this physical derangement, and did its presence in the system so impoverish the blood and thereby the nerve tone as to produce the train of symptoms which occurred throughout the course of the disease? In my own mind there is such a possibility, and yet in studying the literature of any similar case reported I can find nothing which would lead me to such an affirmative answer. I have always been a great believer in thinking that more information is gained by the report of a case than any lengthy article on some abstruse theoretical subject, hence I offer this as my apology for presenting this, to me, an exceedingly interesting case, and will be only too glad to accept the opinion of others as to a correct diagnosis, provided such opinions are based upon a ripe experience and deductions fortified by medical reasoning.

